# COVID-19 and Parkinson’s Disease: Shared Inflammatory Pathways Under Oxidative Stress †

**DOI:** 10.3390/brainsci10110807

**Published:** 2020-10-31

**Authors:** Zahara L. Chaudhry, Donika Klenja, Najma Janjua, Gerta Cami-Kobeci, Bushra Y. Ahmed

**Affiliations:** 1 Institute of Biomedical & Environmental Science and Technology, School of Life Sciences, Faculty of Creative Arts, Technologies & Science, University Square, University of Bedfordshire, Luton LU1 3JU, UK; zohara.chaudhry@beds.ac.uk (Z.L.C.); Gerta.Cami-Kobeci@beds.ac.uk (G.C.-K.); 2 School of Cellular and Molecular Medicine, University of Bristol, University Walk, Bristol BS8 1TD, UK; wo18459@bristol.ac.uk; 3 Faculty of Medicine, Kawasaki Medical School, 577 Matsushima, Kurashiki, Okayama 701-0192, Japan; jann@med.kawasaki-m.ac.jp

**Keywords:** Parkinson’s disease, SARS-CoV-2, caspase, inhibitors, nuclear factor kappa B (NFκB), 6OHDA, oxidative stress, apoptosis

## Abstract

The current coronavirus pandemic caused by the severe acute respiratory syndrome coronavirus 2 (SARS-CoV-2) has resulted in a serious global health crisis. It is a major concern for individuals living with chronic disorders such as Parkinson’s disease (PD). Increasing evidence suggests an involvement of oxidative stress and contribution of NFκB in the development of both COVID-19 and PD. Although, it is early to identify if SARS-CoV-2 led infection enhances PD complications, it is likely that oxidative stress may exacerbate PD progression in COVID-19 affected individuals and/or vice versa. In the current study, we sought to investigate whether NFκB-associated inflammatory pathways following oxidative stress in SARS-CoV-2 and PD patients are correlated. Toward this goal, we have integrated bioinformatics analysis obtained from Basic Local Alignment Search Tool of Protein Database (BLASTP) search for similarities of SARS-CoV-2 proteins against human proteome, literature review, and laboratory data obtained in a human cell model of PD. A Parkinson’s like state was created in 6-hydroxydopamine (6OHDA)-induced differentiated dopamine-containing neurons (dDCNs) obtained from an immortalized human neural progenitor cell line derived from the ventral mesencephalon region of the brain (ReNVM). The results indicated that SARS-CoV-2 infection and 6OHDA-induced toxicity triggered stimulation of caspases-2, -3 and -8 via the NFκB pathway resulting in the death of dDCNs. Furthermore, specific inhibitors for NFκB and studied caspases reduced the death of stressed dDCNs. The findings suggest that knowledge of the selective inhibition of caspases and NFκB activation may contribute to the development of potential therapeutic approaches for the treatment of COVID-19 and PD.

## 1. Introduction

The COVID-19 pandemic caused by the severe acute respiratory syndrome coronavirus 2 (SARS-CoV-2) transmitted from human-to-human has impacted almost each and every corner of the globe and has resulted in a serious health crisis. According to the World Health Organization, as of September 2020, the rapid viral infection transmission has led to more than 960,000 deaths and 31,000,000 cases globally.

A major proportion of COVID-19 cases suffer from severe acute respiratory distress syndrome (ARDS), similar to the disease caused by SARS-CoV and MERS-CoV [1,2]. These similarities could be due to the structural resemblances between the receptor binding domains of SARS-CoV and MERS-CoV, as highlighted by recently published studies showing genomic characterization and epidemiology of SARS-CoV-2 [3,4]. The SARS-CoV-2-induced immunopathogenesis impairs the host immune system, leading to inflammatory responses. The virus enters the cell through its interaction with the angiotensin converting enzyme II (ACE2) receptor and transmembrane serine protease-2 (TMPRSS2) [4,5]. Consequently, the level of Angiotensin 2 (AngII) increases in the serum as a result of its decreased degradation by ACE2. The accumulated AngII has been shown to induce activation of inflammatory cytokines, including IFN-Y, which is then followed by stimulation of interferon (IFN) genes, resulting in an enhanced cytokine storm and the related syndrome ARDS, as observed in severe cases [6,7,8]. Furthermore, cytokine activation leads to hyperactivation of the down-stream signalling cascades including the nuclear transcription factor-kappa B (NFκB), which is normally activated by SARS-CoV-2 itself via pattern recognition receptors (PRRs) [1,7]. Altogether, this comprises a machinery that acts on the co-activation of signal transducer and activator of transcription 1 and 3 (STAT1 and STAT3), which further enhances the activation of NFκB [7,9,10,11,12,13], an inducible transcription factor that has been suggested to trigger apoptotic cell death following oxidative stress and inflammation [14,15,16,17,18,19]. NFκB resides in the cytoplasm in an inactive form in virtually all cell types. Upon activation, NFκB enters the nucleus and promotes transcription of different genes such as those encoding caspases, cytokines, receptors, growth factors, adhesion molecules and chemokines [19,20].

Increased traumatic stress levels seen in SARS-CoV-2 cases may be due to enhanced levels of inflammatory mediators contributing to clinical complications. This may involve activation of pulmonary vasculature endothelial cells leading to the development of hypoxia [2,21]. Respiratory hypoxia seen in COVID-19 patients can also initiate oxidative stress in the brain [22]. It is well known that hypoxia encourages the production of reactive oxidative species (ROS), which are involved in inflammation and immune response, thus influencing cell signalling pathways [22,23]. The elevated levels of ROS can cause redox imbalance, enhance lipid peroxidation products and induce opening of the permeability transition pores of the mitochondria. Due to the imbalance of key electrons in the mitochondria, factors such as pro-caspase, apoptosis initiating factor and cytochrome c are activated [23,24,25]. These factors contribute to further damage of the cell by promoting apoptotic cell death [26,27,28].

There is also accumulating evidence that oxidative stress caused by increased production of ROS following hypoxia contributes to the death of dopamine-containing neurons (DCNs) via apoptosis, leading to the development of Parkinson’s disease (PD), a progressive neurodegenerative disorder [29,30,31]. Patients diagnosed with PD lack DCNs in the *pars compacta* region of the *substantia nigra* (SNpc) and in the *striatum*.

The 6-hydroxydopamine (6OHDA) is a well-known neurotoxin which induces neurotoxicity onto the nigrostriatal dopaminergic system by inhibiting the mitochondrial electron chain complexes I and IV, and promoting degeneration of DCNs [31,32,33,34]. Consequently, it leads to dopamine deficiency and has a profound impact on dopaminergic receptors. Interestingly, the neurotransmitter dopamine and its receptors have been shown to be involved in the regulation of breathing [35,36,37]. Hence, such degeneration may enhance breathing shortness induced by the aforementioned hypoxic condition, and further promote pulmonary impairment [29,36]. Moreover, 6OHDA has been reported to be produced endogenously in PD patients. Thus, in vitro and in vivo models of 6OHDA have been used to mimic the key attributes such as α-synuclein aggregation, iron accumulation and mitochondrial dysfunction to study the advanced stages of PD pathogenesis [38,39,40].

Although intensive research has been conducted, the actual cause of the development and progression of PD is not yet known. Several lines of evidence suggest that DCNs’ death could be a result of elevated levels of ROS [39,40,41], respiratory failure of the mitochondria [42,43,44], and activation of NFκB and caspase pathways [44,45,46]. The role of NFκB in the cell is controversial. A significant increase in NFκB activity has been reported in the SNpc region of the 1-methyl, 4-phenyl, 1, 2, 3, 6-tetrahydropyridine (MPTP)-treated mice [47]. The authors of [47] suggested that degeneration of DCNs could have been prevented by selectively suppressing activation of NFκB. In contrast, activation of NFκB by Parkin through phosphorylation of IκB triggers transcription of pro-survival genes. However, NFκB activation by mutant Parkin promotes cell death [48]. Furthermore, activation of NFκB by 6OHDA in SH-SY5Y neuroblastoma cells has been shown to initiate caspase-3 activation and, as a consequence, death of DCNs through the NFκB pathway [49].

Caspases, a family of cysteine proteases (caspase 1–14), exist in most cells in a dormant state known as zymogens and can be activated through intrinsic or extrinsic routes such as mitochondrial, NFκB and ER stress pathways [50,51,52]. Caspases have been classified as either initiator caspases (e.g., caspase-2, -8, -9 and -10) or effector caspases (e.g., caspase-3, -6 and -7) as upstream and downstream, respectively [53,54]. Initiator caspases such as caspase -2 and -8 are activated via the dimerization process instead of cleavage and can activate effector caspases, resulting in the death of cells via apoptotic routes [53,54].

The development and progression of SARS-CoV-2 and PD are a pool of undefined complex cellular and molecular events. The cell death mechanisms involved in these events are not fully understood. Since oxidative stress is one of the causes of respiratory hypoxia seen in SARS-CoV-2 and PD patients, we aimed to investigate similarities in apoptotic pathways which are activated in response to oxidative stress and their correlation with NFκB activation in both diseases. Understanding the links between oxidative stress, the consequent activation of NFκB and the resulting cellular and molecular responses of downstream genes requires a systematic understanding of the pathways that lead the cells to injuries or apoptotic death. The present study utilizes experimental data, bioinformatics analyses obtained from Basic Local Alignment Search Tool of protein databases (BLASTP) search, and a literature review to investigate NF-κB and caspase activation under SARS-CoV-2-mediated infection and in 6OHDA-treated differentiated DCNs (dDCNs) from an immortalized human neural progenitor cell line derived from the ventral mesencephalon region of the brain (ReNVM) [55,56].

## 2. Materials and Methods

### 2.1. Bioinformatics Analysis: SARS-CoV-2 Infection

Upon cell entry, SARS-CoV-2 releases its RNA genome containing around 14 open reading frames (ORFs), which encode structural or non-structural proteins that contribute to the virus survival and virulence [57]. In this analysis, the sequences of these SARS-CoV-2 ORF-encoded proteins were obtained using the NCBI databases and were then compared with the whole human proteome, using BLASTP search, to look for similarities and to possibly gain further understanding of the function of these viral proteins.

### 2.2. Laboratory-Based Analyses: Antibodies and Inhibitors

#### 2.2.1. Primary Antibodies

Anti-NFκB, p65 subunit (MAB3026), anti-tyrosine hydroxylase (TH) and cleaved anti-caspase-3 (AB3623) were purchased from Millipore, Hertfordshire, UK; anti-caspase-2 (ab7979) and anti-caspase-8 (ab52183) were purchased from Abcam, Cambridge, UK.

#### 2.2.2. Secondary Antibodies

Donkey anti-mouse immunoglobulin G (IgG)-horseradish peroxidase (HRP) goat anti-rabbit IgG-HRP, sheep anti-rabbit IgG-fluorescein isothiocyanate (FITC), sheep-anti-mouse IgG-rhodamine, goat anti-rabbit IgG-rhodamine and donkey anti-mouse IgG-FITC were purchased from Millipore, Hertfordshire, UK.

#### 2.2.3. Inhibitors

Inhibitor of NFκB kinase (IKK; 401479), caspase-2 inhibitor zVDVADfmk (218744), and caspase-8 inhibitor zIETDfmk (218759) were obtained from Merck Chemicals, Nottingham, UK, while universal caspase inhibitor zVADfmk (G7231) was obtained from Promega, Southampton, UK.

### 2.3. Cell Culture

ReNVM cells (Millipore, Watford, UK) were cultured either on laminin-coated T25 tissue culture flasks or chamber slides for Western blot (WB) and immunofluorescence (IF) analyses, respectively. The cells were maintained in ReNVM neural stem cell maintenance medium (SCM005; Millipore, Watford, UK) and were differentiated into dopamine-containing neurons (dDCNs) after withdrawal of epidermal growth factors (EGF) and basic fibroblast growth factor (bFGF). The dopamine marker tyrosine hydroxylase was used to ensure that ReNVM had differentiated into DCNs (dDCNs). To induce stress in all relevant experiments, the dDCNs were treated with 100 µm 6OHDA for 2 h, after which media was replaced with fresh media, and dDCNs were left in the incubator to recover at 37 °C overnight and collected after 24 h as described previously [56]; these cells were termed as 6OHDA-treated dDCNs. The untreated group was subjected to media change only, termed as control.

For further analysis, caspases-2 and -8 inhibitors zVDVADfmk and zIETDfmk, respectively, were used after 6OHDA-induced stress to determine if these inhibitors could protect dDCNs following 6OHDA-mediated toxicity. In addition, NFκB inhibitor, IKK and universal caspase inhibitor zVADfmk were used to determine the involvement of caspases-2 and -8 in the NFκB classical pathway in control and 6OHDA-treated dDCNs. An optimal condition for the IKK treatment (70 µm for 2 h exposure) on dDCNs was determined by treating dDCNs with or without 100 µm 6OHDA for 2 h followed by IKK. To investigate the synergistic effect of IKK with other caspase inhibitors, control and 6OHDA-treated dDCNs were treated with either caspase inhibitors including caspase-2 inhibitor (20 µm zVDVADfmk), caspase-8 inhibitor (80 µm zIETDfmk) and universal caspase inhibitor (50 µm zVADfmk) along with, or without, 70 µm IKK for 2 h. The exposure time of 2 h was kept in coordination with 6OHDA treatment.

### 2.4. Immunocytochemistry

**Figure 1:**
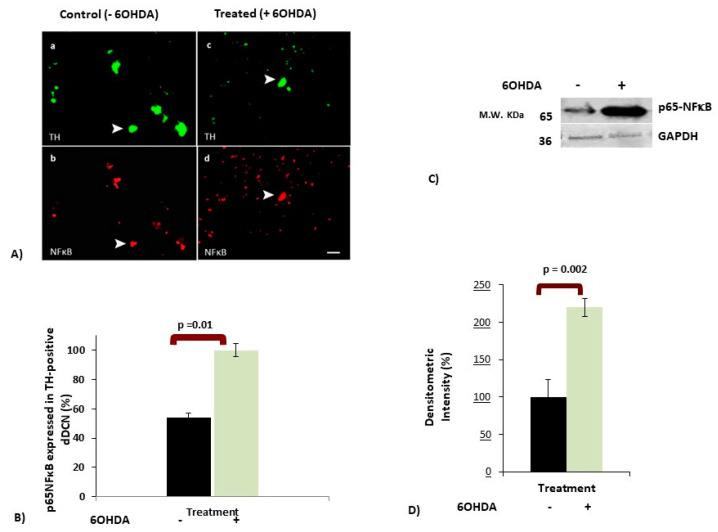
The effect of 6-hydroxydopamine (6OHDA) treatment on NFκB expression in differentiated dopamine-containing neurons (dDCNs). The 6OHDA-treated dDCNs were exposed to 100 µm 6OHDA for 2 h to induce stress, as mentioned in Section 2.4. (**A**) Figure shows positive staining for TH (green), a marker for dopaminergic neurons, in control and 6OHDA-treated dDCNs, indicating that cells are dopamine-containing neurons. Positive staining for the p65-NFκB (red) was found in control and 6OHDA-treated dDCNs. The white arrow indicates positive staining for TH and p65-NFκB in the same dDCN in both control and 6OHDA-treated dDCNs. Scale bars a and b = 50 µm, while c and d = 100 µm. (**B**) The graph illustrates the proportion of the p65-NFκB expressed in TH positive control and 6OHDA-treated dDCNs. Increased expression of the p65-NFκB was observed in dDCNs following exposure to 6OHDA, indicating that 6OHDA-induced toxicity enhanced NFκB activity in dDCNs. Means of three experiments ± SEM are shown, *p* < 0.05. (**C**) The effect of 6OHDA treatment on p65NFκB level was measured and compared with control and 6OHDA-treated dDCNs. Briefly, cell extracts from both control and 6OHDA-treated dDCNs were subjected to Western immunoblotting. Membranes were probed with antibodies for p65NFκB and housekeeping protein GAPDH. (**D**) Densitometric analysis showed a significant increase in p65-NFκB level in dDCNs that were treated with 6OHDA. The (+) and (-) signs indicate with or without treatment respectively. Densitometric value represents mean ± SEM of five experiments. Table of densitometry values and statistical analysis can be found in supplementary Table S1 and S2 for B and D, respectively.

**Figure 2:**
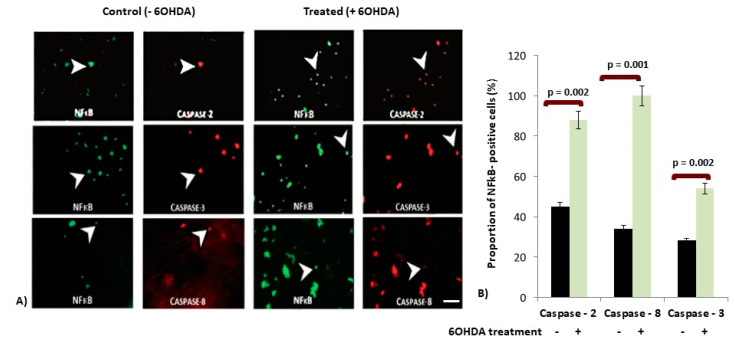
The effect of 6OHDA treatment on the expression of caspases in NFκB-positive dDCNs. (**A**) Figure shows positive staining for p65-NFκB (green) and for caspase-2, -3 and -8(red) in control and 6OHDA-treated dDCNs. Caspase-2 expression was observed in the majority of p65-NFκB positive control dDCNs. A higher expression of caspase-3 was observed in p65-NFκB positive 6OHDA-treated dDCNs when compared to control dDCNs. Caspase-8 was found in low levels in control dDCNs, but an increased expression of caspase-8 was observed in p65-NFκB positive 6OHDA-treated dDCNs. White arrow indicates the positive staining of some dDCNs stained with the p65-NFκB and caspase-2 (upper panel), p65-NFκB and caspase-3 (middle panel) and p65-NFκB and caspase-8 (lower panel) in both control and 6OHDA-treated dDCNs. Scale bar = 100 µm. (**B**) Graph shows the proportion of the p65-NFκB positive cells that expressed caspases-2, -3 and -8 in control and 6OHDA-treated dDCNs. The proportion of caspases-2, -3 and -8 was expressed in less than half of p65-NFκB positive control dDCNs. A significant increased proportion of caspases-2, -3 and -8 in p65-NFκB positive cells was observed after 6OHDA treatment. The (+) and (-) signs indicate with or without treatment respectively. Means of three experiments ± SEM are shown. A table of values and statistical analysis can be found in supplementary Table S3.

Immunocytochemical analysis was used to establish the presence of cleaved p65-NFκB in TH positive dDCNs following 6OHDA treatment. dDCNs were grown and exposed to 100 µm 6OHDA for 2 h as described above. On the next day, control and 6OHDA-treated dDCNs were fixed with 4% paraformaldehyde for 15 min and were washed with cold PBS. Subsequently, cells were treated with 0.1% Triton X-100 (10 min) and blocked with 10% goat serum for 40 min prior to incubation with the primary antibody (anti-TH, 1:1500,) and with cleaved (p65 subunit) anti-NFκB (1:1500) overnight at 4oC, and incubated with the secondary antibody (sheep anti-rabbit IgG-FITC, 1:800) (sheep-anti-mouse IgG rhodamine, 1:300) for 2 h at room temperature. dDCNs were mounted using Vectashield mounting medium and viewed under a Meiji fluorescent microscope (Mazurek, Warwickshire, UK). Furthermore, co-localisation studies were performed to determine if NFκB and TH (see Figure 1), and NFκB and caspases (see Figure 2) were present in the same cell by using the primary antibodies anti-TH (1:1500), anti-NFκB, p65 subunit (1:1500), anti-caspase-3 (1:1000), anti-caspase-2 (1:2000) and anti-caspase-8 (1:2000), and the secondary antibodies donkey anti-mouse IgG-FITC (1:500) and goat anti-rabbit IgG-rhodamine (1:2500). Control and treated dDCNs were counted under × 20 magnification in 5 fields of vision per area (1.428 mm × 1.092 mm). To analyse co-localisation data, cells localised in the same field and stained with two different fluorochromes were counted. Cell numbers are expressed as the mean per selected field from the wells. Three independent experiments were performed for each sample prior to statistical analysis (Student’s *t*-test, *p* < 0.05), as shown in Figure 1B and Figure 2B.

### 2.5. Western Blot Analysis

Control (untreated), 6OHDA-treated, IKK-treated, and 6OHDA + IKK-treated dDCNs were used for WB analyses. Cells were lysed and protein concentrations were measured using the bicinchonic acid (BCA) kit (Pierce Biotechnology, Rockford, IL, USA). Fifty micrograms of protein were loaded on 12% gel prior to SDS PAGE, followed by WB. The list of primary and respective secondary antibodies used in WB analyses is shown in the table below (Table 1):

### 2.6. MTT Assay

The 3-(4,5-dimethylthiazolyl-2)-2,5-diphenyltetrazolium bromide (MTT, Cambridge Bioscience, UK) assay was performed to measure survival of dDCNs following different treatments in control and 6OHDA-treated dDCNs. Briefly, dDCNs were grown in 96 well plates and were left until 80% confluence. dDCNs were treated with different combinations of inhibitors (see Figures for details) along with 6OHDA for 2 h, after which old media was replaced with fresh media and cells were left to recover overnight as described above. The following day, old media was replaced with fresh media prior to the addition of 10 µL of MTT per well. All samples were left to incubate at 37 °C for 4 h. The formazan complex was broken by pipetting the crystals up and down in each well. Subsequently, samples were placed in a microplate reader and the programme Stingray was used to determine the readings of samples at 570 nm wavelength. The readings were normalised prior to statistical analysis at *p* < 0.05 using ANOVA and Student’s t-test. Three independent experiments were conducted each for untreated, 6OHDA-treated and inhibitor(s)-treated dDCNs.

### 2.7. TUNEL Assay

To explore if 6OHDA stimulates death of dDCNs via apoptotic or necrotic pathways, the terminal deoxynucleotidyltransferase (TdT)-mediated 2′-deoxyuridine 5′-triphosphate nick end-labelling assay (TUNEL) in control and 6OHDA-treated dDCNs was performed as recommended by the manufacturer (Trevigen, MD, USA). Various inhibitors, such as IKK, zVADfmk, zVDVADfmk and zIETDfmk, in the presence and/or absence of 6OHDA, were also used. The proportion of apoptotic dDCNs was determined by TUNEL absorbance at 450 nm.

### 2.8. Detection and Statistical Analysis

Band analysis was carried out using a densitometer (GS800, Bio-Rad, Hertfordshire, UK). Blots were scanned using GS800 scanner, the band lanes and bands were detected automatically using Quantity One software at default settings, and the raw densitometric intensity values were obtained. The housekeeping protein Gylceraldehyde-3-Phosphate Dehydrogenase (GAPDH) was used as a reference guide and the densitometric values for the experimental samples were normalised against GAPDH to determine the densitometric ratio prior to carrying out statistical analysis (Student’s t-test, *p* < 0.05, ANOVA, *p* < 0.05). All results in graphs are shown as relative percentage compared with control to provide better comparison between control and 6OHDA-treated dDCNs.

Three to five independent experiments were performed for each treatment and values are indicated in ± SEM. Table of values and statistical analysis can be found in supplementary data; the relevant number of experiments performed is mentioned in the figure legends.

## 3. Results

### 3.1. SARS-CoV-2 Infection Induces Pro-Apoptotic Responses

Significant matches obtained from BLASTP search involved the viral polyprotein pp1ab, which resembled two human proteins PARP14 and PARP9, as shown in Table 2. Viral pp1ab originates from the genome regions that encode non-structural proteins (Nsps), ORF1a and ORF1b, which are translated into two overlapping polyproteins, pp1a and pp1ab, via a ribosomal shifting event. These polyproteins are further cleaved through encoded proteases into 1–11 Nsps (pp1a) and 1-16 Nsps (pp1ab) [57]. Results demonstrate that pp1ab shares 32% identities and 49% positives with human protein mono-ADP-ribosyl transferase PARP14 (E-value: 3 × 10^−6^) at the 1056–1169 position of pp1ab, whereas at the 1057–1186 position pp1ab shares 31% identities and 45% positives with human protein mono-ADP-ribosyl transferase PARP9 (E-value: 2 × 10^−4^) (Table 2).

The positions of SARS-CoV-2 pp1ab, that resemble human PARP isoforms, encompass the Nsp3 viral non-structural protein region. This is a large multifunctional protein comprising various domains, which are differently organised across CoV genera. Interestingly, this region of similarity is shown to precisely cover the ADP-ribose-binding module in position 238–337 of Nsp3, part of the conserved Nsp3 macrodomain X, which is known to remove mono-ADP-ribose from many targets [12,57]. The similarity observed between viral Nsp3 and human PARP9 specifically encompasses a region of PARP9 that corresponds to Macrodomain 1 located in position 107–296 of PARP9 (UniProt). These results suggest important information about the function of the viral Nsp3, when considering its resemblance with PARP human isoforms, as discussed later.

### 3.2. 6OHDA-Induced Toxicity Amplifies Activation of NFκB in dDCNs

To investigate if 6OHDA-induced stress in dDCNs follows the NFκB pathway, double immunocytochemistry (ICC) analysis using NFκB and tyrosine hydroxylase (TH) was performed. Results showed the presence of p65-NFκB in control and 6OHDA-treated dDCNs (Figure 1A). The presence of p65-NFκB in control dDCNs is not surprising as NFκB is a diverse transcription factor required for several cellular functions. However, the control dDCNs showed a lower expression of p65-NFκB than that of the TH (Figure 1A, left panel). This could simply reflect a difference in sensitivity between TH and NFκB antibodies; although, the exposure of dDCNs to 6OHDA increased the number of p65-NFκB positive cells (Figure 1A, right panel), indicating activation of NFκB following 6OHDA-induced stress.

Quantitative analysis (see Section 2.4; Immunocytochemistry for detail) showed a difference in the number of p65-NFκB cells expressed in TH-positive 6OHDA-treated and control dDCNs. P65-NFκB was expressed in 54% of TH-positive control dDCNs. However, 6OHDA-induced stress significantly increased (46%) the proportion of p65-NFκB in dDCNs, illustrating that 6OHDA triggers activation of NFκB in dDCNs (Figure 1B). Western blot (WB) analysis also revealed a substantial increase (122%) in the amount of p65-NFκB in 6OHDA-treated dDCNs compared to control dDCNs (Figure 1C,D), supporting the notion that 6OHDA-induced stress triggers NFκB activation.

### 3.3. Increased Expression of Caspases in p65-NFκB Expressed dDCNs Following 6OHDA-Induced Stress

Here, we sought to explore the involvement of NFκB in caspase pathways triggered by 6OHDA-induced stress in dDCNs. Co-localisation studies were carried out to determine if specific caspase along with p65-NFκB were present in both control and 6OHDA-treated dDCNs. Increased expression of p65-NFκB and caspases-2, -3 and -8 were observed in the dDCNs following 6OHDA treatment (Figure 2A). Quantitative analysis (see Section 2.4; Immunocytochemistry for details) revealed a positive correlation between caspases-2, -3, -8 and p65-NFκB in 6OHDA-treated dDCNs. Statistical analysis was performed using ANOVA and Student’s t-test to measure the statistical differences in the proportion of p65-NFκB positive cells that express caspase along with p65-NFκB in dDCNs before and after exposure to 6OHDA (Figure 2B). Control p65-NFκB-positive dDCNs expressed 45%, 31% and 34% caspases-2, -3 and -8, respectively. However, 6OHDA treatment increased the expression of caspases-2, -3 and -8 to 88%, 54% and 100%, respectively. These results indicate that caspases-2, -3 and -8 are involved in NFκB-mediated death in 6OHDA-treated dDCNs, suggesting involvement of NFκB pathway(s) in the death of dDCNs.

### 3.4. IKK Suppressed Activation of p65-NFκB in 6OHDA-Treated dDCNs

**Figure 3:**
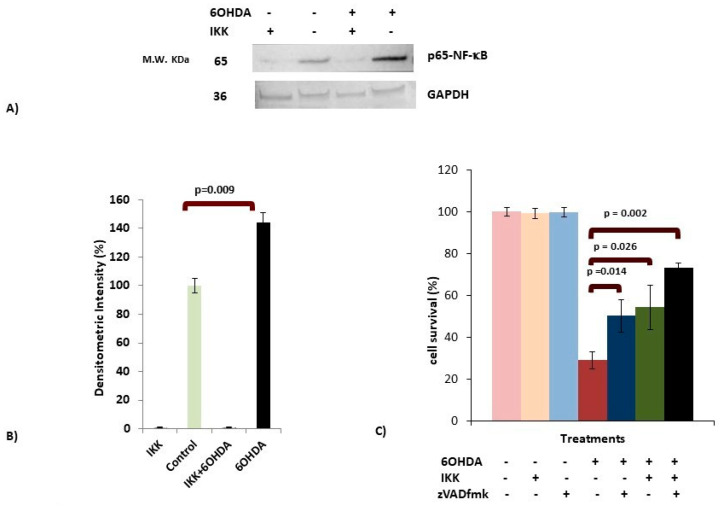
6OHDA stimulates the classical NFκB pathway in dDCNs. (**A**) Control and 6OHDA-treated dDCNs were treated with 70 μM Inhibitor of NFκB kinase (IKK for 2 h (see Section 2.3). Cell extracts were subjected to WB immunoblotting and membranes were probed with p65-NFκB and Gylceraldehyde-3-Phosphate Dehydrogenase (GAPDH) antibodies. The absence of NFκB was observed in IKK-treated dDCNs, suggesting that the NFκB classical pathway is involved in death of dDCNs. Illustrative examples of p65-NFκB and housekeeping protein GAPDH in control, IKK, 6OHDA, IKK + 6OHDA-treated dDCNs are shown. (**B**) Densitometric analysis showed a significant increase in p65-NFκB levels in 6OHDA-treated dDCNs compared to control dDCNs (*p* < 0.01). (**C**) Control and 6OHDA-treated dDCNs were treated with zVADfmk and IKK. Results showed that combined treatments significantly decreased death of 6OHDA-treated dDCNs. However, both inhibitors synergistically could not provide complete inhibition of cell death induced by 6OHDA toxicity in dDCNs. The proportion of cells surviving was determined by MTT absorbance at 570 nm. The (+) and (-) signs indicate with or without treatment respectively. Means of three experiments ± SEM are shown in B and C. A table of densitometry values and statistical analysis can be found in supplementary Tables S4 and S5 for B and C, respectively.

The classical, alternative and atypical pathways stimulate NFκB activation. To determine if 6OHDA-induced stress triggered the NFκB classical pathway in dDCNs, an IKK-inhibitor 401479, which specifically suppresses the NFκB classical pathway, was used. IKK inhibited activation of p65-NFκB in 6OHDA-treated dDCNs, demonstrating that 6OHDA-induced stress stimulates the NFκB classical pathway. WB analysis showed 44% increased expression of p65-NFκB in 6OHDA-treated dDCNs. However, the absence of NFκB in 6OHDA + IKK-treated dDCNs compared with control signified the involvement of the NFκB classical pathway in the death of dDCNs (Figure 3A,B).

To find if 6OHDA-induced stress can trigger additional pathways other than the NFκB classical pathway that results in death of dDCNs, a combined effect of universal caspase inhibitor zVADfmk and a specific NFκB inhibitor IKK was used. zVADfmk and IKK significantly increased the survival of 6OHDA-treated dDCNs (50% and 54%, respectively) compared to 29% in 6OHDA-treated dDCNs. A further increase in cell survival was found when 6OHDA-treated dDCNs were treated with zVADfmk along with IKK (73%), showing the synergistic effect of both inhibitors. However, both inhibitors were unable to provide 100% protection to 6OHDA-treated dDCNs, indicating that other caspase-related pathway(s) might be contributing towards the death in 6OHDA-treated dDCNs (Figure 3C).

### 3.5. OHDA Provokes Apoptotic Death in dDCNs

**Figure 4:**
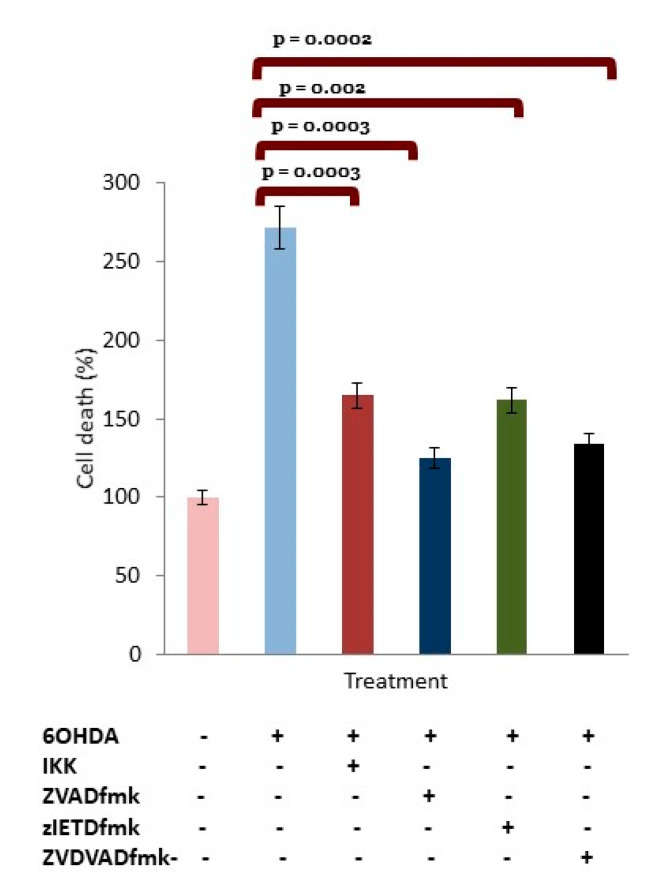
6OHDA triggered apoptotic death in dDCNs. To determine if 6OHDA stimulated death of dDCN is via the apoptotic or necrotic route, the TUNEL assay was used. Various inhibitors such as IKK, zVADfmk, zVDVADfmk, and zIETDfmk were used with 6OHDA to determine if the inhibitor decreased 6OHDA-mediated death of dDCNs. 6OHDA triggered death of dDCNs via the apoptotic route. The result illustrates that all studied inhibitors reduced apoptotic death of dDCNs at various rates. The result is shown as the relative percentage of cell death compared with control. The (+) and (-) signs indicate with or without treatment respectively. Means of three experiments ± SEM are shown. A table of values and statistical analysis can be found in supplementary data S6.

The subsequent aim was to determine if 6OHDA-induced stress in dDCNs follows necrotic or apoptotic pathways. To achieve this, the Tunnel assay was performed. The results showed that 6OHDA treatment significantly encouraged death of dDCNs through an apoptotic pathway. ANOVA and Student’s t-test analysis (*p* < 0.05) revealed a significant difference in the level of apoptotic death in 6OHDA-treated dDCNs (272%). Apoptotic death of dDCNs confirms that 6OHDA-induced oxidative stress triggers caspase activation, resulting in the death of dDCNs via the apoptotic route (Figure 4).

## 4. Discussion

The results from our bioinformatics analysis show a similarity between SARS-CoV-2 Nsp3 and human PARP14 (Table 2a). Human PARP14 is a protein involved in the STAT1 mono-ADP-ribosylation process [58]. STAT1 is particularly important in the IFN receptor signalling pathway where it forms homo or heterodimers, following its phosphorylation at tyrosine position 701 (STAT1 Tyr701). This contributes to the formation of downstream factors that translocate to the nucleus and in turn affect gene expression. Mono-ADP ribosylation of STAT1 by PARP14 impedes STAT1 phosphorylation and, as a consequence, downregulates the responses triggered by interferons (IFNs) [58]. Recent evidence [12] shows matching protein sequence similarity results with correspondence to PARP14 when comparing SARS-CoV-2 Nsp3 protein against the human proteome using BLASTP, while we compared all SARS-CoV-2 ORF-encoded proteins against the human proteome using BLASTP, and found the significant similarities between pp1ab and PARP human isoforms that resulted to encompass the Nsp3 region of the viral pp1ab. Considering that the SARS CoV-2 Nsp3 macrodomain shares similarity with PARP14, yet both are known to perform opposing functions with regard to ADP-ribose removal/addition, it has been suggested that the two possess similar conformation and compatibility for the removal (Nsp3) and addition of ADP-ribose (PARP14) in STAT1. Thus, in an IFN-activated cell, expressed viral Nsp3 will counteract mono-ADP-ribosylation of STAT1 and therefore upregulate STAT1 phosphorylation [12]. Our evidence of pp1ab similarity with PARP9, although less significant than with PARP14, implies that Nsp3 shares sequence similarity with the Macrodomain 1 region of PARP9 (Table 2b), which is dispensable for interaction with STAT1 and suppression of PARP14-mediated STAT1 ADP-ribosylation [58,59]. This further supports the potential role of Nsp3 in promoting STAT1 phosphorylation in response to IFN-γ stimulation, and therefore prolonging STAT1-dependent expression of IFN-stimulated genes. This prolonged expression can potentially engender the several consequences corresponding with the severity of COVID-19, including enhanced inflammation/cytokine storm as explained by Clevery [12]. Further to this, we investigated the role of promoted STAT1 phosphorylation in enhanced pro-apoptotic responses. The latter is harmonious with the literature findings demonstrating that prolonged activated STAT1 tips the cell survival balance toward IFN-γ-induced apoptosis. This is shown to be mediated by the upregulation of caspase-8 in particular [60,61], and partially caspases-2,-3 and -7 [62]. Regarding caspase-8, there is evidence indicating that its upregulation is signalled through STAT1 and IFN γ inducible interferon regulatory factor 1 (IRF1) enhanced transcription, or by STAT1 independently. Hence, increased STAT1 phosphorylation in the presence of viral Nsp3 protein may lead to increased caspase-8 expression, thus promoting apoptosis initiation by the cleavage of the downstream effector caspases [60]. Investigations have also shown that STAT1 is important in regulating the constitutive mRNA level of caspase-2 and thus promoting apoptosis [62]. Another recent study further supports the involvement of caspase-8 in SARS-CoV-2 induced apoptosis [63] implying that ORF3a, a cell membrane-associated viral protein, induces the cleavage/activation of caspase-8, which then acts to convert B-cell lymphoma 2 homology domain 3 interacting-domain (BID) to truncated p15 BID (tBID) [63]. Subsequently, tBID binds to the pores of the mitochondria, thereby promoting the opening of mitochondrial PTP triggering cytochrome c release, followed by apoptosome formation and caspase-9 activation, which in turn activates effector caspase-3 to promote cell destruction [44,63].

Although we have not studied the STAT1 phosphorylation process in our experimental PD model, there is a wealth of information showing the activation of STAT1 following hypoxia, leading to inflammatory response activated by IFN-γ, gangliosides and NFκB [64,65,66,67]. Oxidative stress caused by respiratory hypoxia also activates NFκB and has a negative impact on the function of DJ-1 (Parkinson protein 7-PARK7). Mutations of the latter have been identified in early onset autosomal recessive PD. Several investigations suggest that loss of PARK7 function may increase the PD risk through enhanced brain inflammation. Considering that DJ-1 normally functions to prevent prolonged STAT1 activation, loss of its function is shown to lead to increased STAT1 phosphorylation and upregulated inflammatory mediators in response to IFN-γ, similar to SARS-CoV-2 infection [65]. Likewise, increased STAT1 phosphorylation will potentially result in caspase-2 and -8 activation, as well as NFκB activation [9,10,11,60,62]. A mass destruction of DCNs caused by prolonged caspase-activation can contribute to the onset of PD [44,68], while the cleavage of BiD and the translocation of its truncated form, tBid, to the mitochondria in PD brain has also been reported [69]. This similarity provides a mechanistic link between development of PD and SARS-CoV-2, which can be further supported by the laboratory results of the present study, showing increased expression of p65-NFκB and caspases-2, -3 and -8 in 6OHDA-treated dDCNs, thus stimulating the NFκB pathway and promoting apoptotic death.

Work by Masumoto et al. [70] had found that NFκB overactivity was encouraged by elevated levels of the apoptosis-associated speck-like protein containing caspase-recruitment domain (ASC). Elevated levels of caspase-8 and NFκB p65 were determined in cells treated with ASC, indicating an overexpression of ASC promoted apoptotic cell death through NFκB and caspase-8 mechanisms. IKK inhibited NFκB p65 and caspase-8 apoptotic death of DCNs that had been exposed to ASC and a member of the Apoptosis-activating family-1 (Apaf-1). In addition, Ye et al. [71] and Xiang et al. [49] have shown 6OHDA triggered phosphorylation of IκBand translocation of p65 to the nucleus in SH-SY5Y neuroblastoma cells. ICC and WB analysis revealed that 6OHDA triggered caspase-3 activation and death of DCNs through the NFκB classical pathway [49,71]. These findings are in parallel with the current study showing that 6OHDA triggers activation of the NFκB classical pathway leading to caspase-mediated apoptotic death of dDCNs. In addition, our results using NFκB, caspases-2 and -8 specific inhibitors demonstrated that NFκB stimulates caspases-2 and -8 expression in dDCNs treated with 6OHDA (Figure 4).

**Figure 5:**
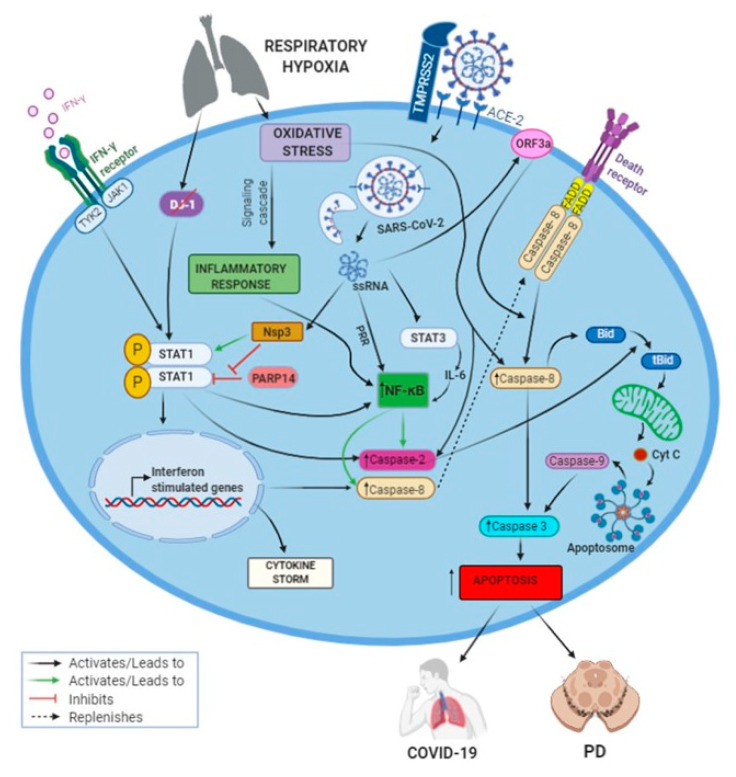
Illustration describing inflammation and apoptosis mechanisms in SARS-CoV-2 infection, suggesting similarity with those in Parkinson’s disease under oxidative stress. The SARS-CoV-2 entry in the cell requires interaction with the surface molecules angiotensin converting enzyme 2 (ACE2) and transmembrane serine protease 2 (TMPRSS2), leading to activation of NFκB via pattern recognition receptors (PRR), which is further amplified by STAT-1, STAT-3 and oxidative stress, similar to 6OHDA-induced toxicity in treated dDCNs showing increased p65-NFκB expression. This NFκB stimulation induces caspase-2 and -8 expression in 6OHDA-treated dDCNs, which are the upregulated caspases from prolonged STAT-1 activation, NFκB and membrane-associated ORF3a viral protein in SARS-CoV-2 infected cells, leading to apoptotic cell death through the extrinsic pathway, thus promoting the development of PD and COVID-19. (BioRender software was used to create this figure).

Research by Lamkanfi et al. [72] showed that caspase-2 can trigger NFκB activation resulting in caspase-3 activation and leading to the death of cells. Caspase-2 forms a complex with P53-induced death domain and receptor interacting protein which promotes NFκB stimulation. However, our results suggest that NFκB promotes expression of caspase-2 in 6OHDA-treated dDCNs (Figure 2). Similarly, as seen in SARS-CoV-2 infection, where caspase-8 is important in inducing cell apoptosis (Figure 5), inhibition of caspase-8 activity has been shown to reduce death of cortical neurons [73]. Interestingly, increased caspase-8 level in post-mortem brain of LRRK-2-associated PD patients has also been reported [73]. This indicates that mutated LRRK-2 promotes caspase-8 dependent death of striatal DCNs in the progression of PD. In comparison, our experimental results clearly demonstrate that NFκB stimulates expression of caspases-2 and-8, leading to apoptotic death in 6OHDA-treated dDCNs (Figure 2). Ho et al. [73] studied specific PD brain samples that had been associated with a mutated LRRK-2 gene, and therefore, in their study only the effect of mutated LRRK-2 gene on caspase could be determined. Our study, on the other hand, focused on specific caspases and their expression after administration of 6OHDA, and explored the environmental rather than the genetic aspects of the damage that led to the death of dDCNs.

DeErausquin et al. [74] have shown increased levels of NFκB and calcium along with ROS production and decreased IkB expression in rat mesencephalic cultures that were exposed to the glutamate receptor AMPA. ICC analysis portrayed a significant loss of dendrites in DCNs that were treated with AMPA, indicating that excitotoxicity might have promoted the stimulation of NFκB, which in turn led to the death of DCNs. Thus, our results are consistent with the previously published studies demonstrating that increased production of ROS triggers the expression of caspase-3 via the NFκB route, leading to the death of cells through the apoptotic pathway [14,54,75]. In other words, the oxidative stress in both conditions, whether induced by 6OHDA-induced toxicity or mediated by SARS-CoV-2 infection, shares at least one common inflammatory pathway(s) triggering NFκB activation. It is worth noting that caspases-2 and -8 are activated via STAT1 signalling, independent of the NFκB pathway [60,61,62]. However, since in the present study, NFκB stimulation triggered caspases-2 and -8 expressions in 6OHDA-treated dDCNs, it is tempting to assume that NFκB activation may lead to activation of these caspases in SARS-CoV-2 infection as well, causing apoptotic cell death and consequently promoting the development of PD and COVID-19 (Figure 5).

## 5. Conclusions

Taken together, our study of the NFκB signalling pathway in the experimental PD model in comparison with bioinformatics analysis of SARS-CoV-2, supports the notion that activation of the NFκB signalling cascade may be a common inflammatory pathway observed in the pathogenesis of both PD and COVID-19. Our results suggest that manipulation of this pathway and/or selective inhibition of caspase and NFκB activations through different channels may provide better protection to the affected cells under stressed conditions. As NFκB is involved in a diverse range of processes that regulate cellular function in healthy cells, a wide-ranging inhibition of its pathways may contribute to or cause disruptions at the cellular and physiological levels. Further research involving treatments with pharmacological agents and identification of key proteins involved in these pathways may identify potential targets to safely suppress NFκB and caspase activations in stressed cells without impacting other cellular mechanisms, and can open new windows into potential therapeutic approaches to the treatment of COVID-19 and PD. It is too early at this point to recognise whether SARS-CoV-2 exposure will have any long term impact on the development or progression of PD. However, it is likely that the similarities seen between both disorders may add additional weight to an enhanced risk or progression of PD following SARS-CoV-2 infection and/or vice versa in the elderly.

## Figures and Tables

**Table 1:** A list of primary and secondary antibodies used in Western blot analyses.

Primary Antibody	Secondary Antibody
anti-NFκB-p65 (1:5000)	donkey anti-mouse IgG-HRP (1:6000)
anti-caspase-2 antibody (1:2500)	goat anti-rabbit IgG-HRP (1:1000)
anti-caspase-8 antibody (1:1000)	goat anti-rabbit IgG-HRP (1:1000)

**Table 2:**
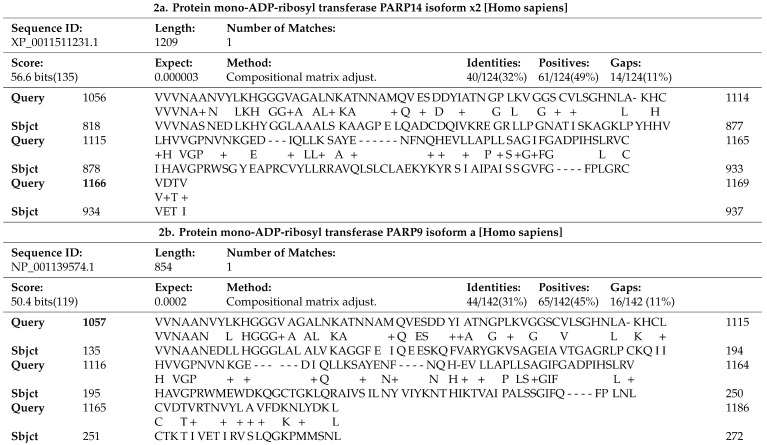
Basic Local Alignment Search Tool of Protein Databases BLASTP search results of SARS-CoV-2 pp1ab similarity with human protein mono-adenosine diphospate (ADP)-ribosyl transferase 14,PARP14 (Alternative name: Poly [ADP-ribose] polymerase 14) (2a) and with human protein mono-adenosine diphosphate (ADP)-ribosyl transferase 9,PARP9 (Alternative name: Poly [ADP-ribose] polymerase 9) (2b). Query refers to the protein sequence of pp1ab and sbjct refers to the protein sequence of PARP9 or PARP14 correspondingly.

## Supplementary Materials

The following are available online at https://www.mdpi.com/2076-3425/10/11/807/s1, Table S1: Determining the proportion of p65 NFkB expressed in TH-positive control (untreated) and 6OHDA-treated dDCNs, Table S2: 6OHDA increased activation of NFκB in dDCNs, Table S3: Determining the proportion of Caspases-2, -3 and -8 expressed in NFκB positive control and 6OHDA-treated dDCNs, Table S4: NFkB is suppressed by IKK in 6OHDA-treated dDCNs, Table S5: IKK and zVADfmk promote survival of 6OHDA-treated dDCNs, Table S6: 6OHDA triggered apoptotic death in dDCNs.
