# Supplementary material for: COVID-19 and Parkinson’s Disease: Shared Inflammatory Pathways Under Oxidative Stress
Source: Brain Sci. 2020 Oct 31;10(11):807. doi: 10.3390/brainsci10110807 (PMC7693814; doi:10.3390/brainsci10110807)
Supplement: Supplementary file 1 [file brainsci-10-00807-s001.pdf]

Supplementary Materials

Table S1: Determining the proportion of p65 NFkB expressed in TH-positive control (untreated) and 6OHDA-treated dDCNs.

	Control (- 6OHDA)		Treated (+6OHDA)	
	**TH**	**NFkB**	**TH**	**NFkB**
Mean of Assay 1	34.60	17.06	9.67	10.00
Mean of Assay 2	36.13	18.22	10.13	11.00
Mean of Assay 3	32.73	20.36	11.93	10.60
Mean of All Assays	34.49	18.55	10.58	10.53
Mean of All Assays %	100.00	53.78	30.67	30.54
Total Ratio	0.54		1.00	
**Total Ratio (%)**	**54**		**100**	
***t*-Test**		**0.01**		

Table 2. 6OHDA increased activation of NFκB in dDCNs.

	**MEAN** Control	**MEAN** Treated (+6OHDA)
NFκB Assay1	0.824	1.360
NFκB Assay 2	0.686	1.217
NFκB Assay3	0.955	1.349
NFκB Assay4	0.363	1.438
NFκB Assay 5	0.310	1.542
Mean Assay	0.627	1.381
**Mean Assay (%)**	**100**	**220**
SD	0.28	0.12
SD (%)	28.31	11.99
***t*-Test**		**0.002**

Table S3: Determining the proportion of Caspases-2, -3 and -8 expressed in NFκB positive control and 6OHDA-treated dDCNs

	Control (-6OHDA)		6OHDA Treated		Control (-6OHDA)		6OHDA Treated		Control (-6OHDA)		6OHDA Treated	
	NFkB	Caspase-2	NFkB	Caspase-2	NFkB	Caspase-3	NFkB	Caspase-3	NFkB	Caspase-8	NFkB	Caspase-8
**Mean of Assay 1**	36.67	19.33	10.00	8.13	41.93	11.00	10.47	5.47	19.67	7.60	9.87	9.87
**Mean of**	42.33	16.01	8.00	8.33	41.40	11.20	9.93	6.00	24.20	8.21	10.40	10.40

Assay 2												
Mean of Assay 3	41.93	18.56	10.87	8.80	39.67	12.00	10.53	5.27	26.40	8.13	12.00	12.00
Mean of All Assays	40.31	17.97	9.62	8.42	41.00	11.40	10.31	5.58	23.42	7.98	10.76	10.76
Mean of All Assays %	100.00	44.57	23.87	20.89	100.00	27.80	25.15	13.60	100.00	34.03	45.92	45.92
Total Ratio	0.45		0.88		0.31		0.54		0.34		1.00	
**Total Ratio (%)**	**45**		**88**		**28**		**54**		**34**		**100**	
***t*-Test**	**0.02**				**0.02**				**0.01**			

Table S4: NFkB is suppressed by IKK in 6OHDA-treated dDCNs

	MEAN Control	MEAN IKK	MEAN 6OHDA-treated	MEAN 6OHDA & IKK-treated
NFκB Assay 1	0.918	0.000	1.488	0.000
NFκB Assay 2	1.069	0.000	1.572	0.000
NFκB Assay 3	1.148	0.000	1.444	0.000
Mean Assay	1.045	0.000	1.502	0.000
**Mean Assay(%)**	**100**	**0**	**144**	**0**
SD	0.12	0.00	0.07	0.00
SD (%)	11.69	0.00	6.50	0.00
***t*-Test**			**0.009**	

Table S5: IKK and zVADfmk promote survival of 6OHDA-treated dDCNs

	**MEAN Control**	**MEAN IKK**	**MEAN zVADfmk**	**MEAN 6OHDA-treated**	**MEAN 6OHDA & zVADfmk**	**MEAN 6OHDA & IKK**	**MEAN 6OHDA & zVADfmk & IKK**
Mean of Assay 1	2.059	2.035	2.103	0.535	1.004	1.285	1.539
Mean of Assay 2	2.121	2.099	2.051	0.671	0.955	1.203	1.545
Mean of Assay 3	2.114	2.109	2.123	0.631	1.205	0.934	1.539
Mean of All	2.098	2.081	2.092	0.612	1.055	1.141	1.541

Assays							
Mean of All Assays %	**100**	**99**	**100**	**29**	**50**	**54**	**73**
SD	0.03	0.04	0.04	0.07	0.13	0.18	0.00
(n)	3	3	3	3	3	3	3
SQR	1.73	1.73	1.73	1.73	1.73	1.73	1.73
SE	0.02	0.02	0.02	0.04	0.08	0.11	0.00
SE %	1.97	2.31	2.15	4.04	7.65	10.60	0.20
***t*-Test**					**0.014**	**0.026**	**0.002**

Table S6: 6OHDA triggered apoptotic death in dDCNs

	**MEAN Control**	**MEAN 6OHDA**	**MEAN 6OHDA & IKK**	**MEAN 6OHDA & zVADfmk**	**MEAN 6OHDA & zIETDfm k**	**MEAN 6OHDA & zVDVADfmk**
Mean of Assay 1	0.79	2.15	1.36	0.98	1.26	1.04
Mean of Assay 2	0.82	2.19	1.27	0.99	1.30	1.06
Mean of Assay 3	0.77	2.10	1.28	1.00	1.27	1.08
Mean of All Assays	0.79	2.15	1.31	0.99	1.28	1.06
**Mean of All Assays%**	**100**	**272**	**165**	**125**	**162**	**134**
SD	0.025	0.045	0.049	0.011	0.02	0.02
(n)	3	3	3	3	3	3
SQR	1.73	1.73	1.73	1.73	1.73	1.73
SE	0.013	0.027	0.027	0.006	0.011	0.010
SE %	1.343	2.711	2.689	0.611	1.069	0.976
***t*-Test**			**0.00003**	**0.0003**	**0.0002**	**0.0002**
